# Resting State fMRI: A Valuable Tool for Studying Cognitive Dysfunction in PD

**DOI:** 10.1155/2018/6278649

**Published:** 2018-04-23

**Authors:** Kai Li, Wen Su, Shu-Hua Li, Ying Jin, Hai-Bo Chen

**Affiliations:** ^1^Department of Neurology, Beijing Hospital, National Center of Gerontology, No. 1 Dahua Road, Dong Dan, Beijing 100730, China; ^2^Department of Geriatric Medicine, Beijing Hospital, National Center of Gerontology, No. 1 Dahua Road, Dong Dan, Beijing 100730, China

## Abstract

Cognitive impairment is a common disabling symptom in PD. Unlike motor symptoms, the mechanism underlying cognitive dysfunction in Parkinson's disease (PD) remains unclear and may involve multiple pathophysiological processes. Resting state functional magnetic resonance imaging (rs-fMRI) is a fast-developing research field, and its application in cognitive impairments in PD is rapidly growing. In this review, we summarize rs-fMRI studies on cognitive function in PD and discuss the strong potential of rs-fMRI in this area. rs-fMRI can help reveal the pathophysiology of cognitive symptoms in PD, facilitate early identification of PD patients with cognitive impairment, distinguish PD dementia from dementia with Lewy bodies, and monitor and guide treatment for cognitive impairment in PD. In particular, ongoing and future longitudinal studies would enhance the ability of rs-fMRI in predicting PD dementia. In combination with other modalities such as positron emission tomography, rs-fMRI could give us more information on the underlying mechanism of cognitive deficits in PD.

## 1. Introduction

Parkinson's disease (PD) is one of the most common neurodegenerative diseases. Traditionally, it has been regarded as a movement disorder characterized by the motor symptoms, such as bradykinesia, resting tremor, and rigidity. Up to now, it is well known that cognitive impairment is a common nonmotor symptom in patients with PD, even in the early stages or before motor symptom onset [1]. Furthermore, about 40% of the PD patients suffer from PD dementia (PDD) in cross-sectional studies [2]. In a longitudinal study, 83% of the PD patients developed dementia during the 20-year follow-up [3]. Despite the heavy burden caused by cognitive impairments in PD, we still lack effective treatments for cognitive symptoms in PD. Although acetylcholinesterase inhibitors could provide modest help, the progression of cognitive decline is inevitable [4].

The underlying mechanism of motor symptoms of PD is depleted dopaminergic cells in the substantia nigra [5]. In contrast, the pathophysiological basis of cognitive impairments in PD remains uncertain. Disrupted frontal-subcortical circuits due to dopaminergic neuron damage and wide deposition of *α*-synuclein, *β*-amyloid, and tau proteins might play a role [6, 7]. Various neurotransmitters including dopamine, acetylcholine, serotonin, and noradrenaline are involved [7]. A better understanding of the pathophysiology of cognitive impairments in PD can facilitate early identification and improved intervention for cognitive symptoms.

Functional MRI (fMRI) measures the blood-oxygen-level dependent (BOLD) signal in the brain, which is determined by the amount of oxyhemoglobin and deoxyhemoglobin and reflects neuronal activity. Resting state fMRI (rs-fMRI) estimates the brain BOLD signal while the subjects are awake without performing any specific task [8]. MRI is widely equipped by hospitals and research institutions; rs-fMRI is easy to perform and has an excellent safety profile compared to other imaging modalities such as computed tomography (CT), positron emission tomography (PET), and single photon emission computed tomography (SPECT). Therefore, the application of rs-fMRI in neurological and psychiatric disorders has been rapidly increasing in the recent two decades. There are many approaches that can analyze the rs-fMRI data, including amplitude of low-frequency fluctuations (ALFF) and regional homogeneity (ReHo) which reflect local activity of individual regions or voxels, as well as seed-based functional connectivity (FC), independent component analysis (ICA), effective connectivity, machine learning, and graph theory-based analyses which measure the connectivity characteristics of different regions [8, 9]. rs-fMRI has been applied to investigate the pathophysiology of motor and nonmotor symptoms in PD, help early and differential diagnosis, predict disease progression, and guide treatment. In this review, we summarize recent developments of rs-fMRI studies on cognitive impairments in PD.

## 2. Uncovering the Pathophysiology of Cognitive Impairment in PD Using rs-fMRI

### 2.1. rs-fMRI Studies on PD Patients with Mild Cognitive Impairment (MCI) or Dementia

Cognitive activities rely on the coordination of various brain regions. Several networks have been established by rs-fMRI: the default mode network (DMN), the visual network, the sensorimotor network, the executive control network, and the frontoparietal network [10]. rs-fMRI is useful for improving our understanding of the mechanism of cognitive impairment in PD. Gorges et al. performed seed-based analyses on rs-fMRI in PD patients with and without MCI and healthy controls. Compared with the controls, PD patients without cognitive impairment had increased FC in multiple regions, while PD-MCI patients had decreased FC mainly within the DMN. The increased FC in PD patients without cognitive impairment might be a compensatory mechanism preceding PD-MCI [11]. Hou et al. conducted a study on drug-naïve PD patients with and without MCI and found FC reduction in both PD groups. In addition, compared with PD patients without cognitive impairment, PD-MCI patients had significantly reduced FC between DMN and the middle frontal and middle temporal gyri; within the DMN, PD-MCI patients had reduced FC between the anterior temporal lobe and inferior frontal gyrus. The FC alterations in the PD group were associated with attention/working memory and memory function [12]. Chen et al. studied the FC between posterior cingulate cortex and other regions of the bran in PD patients with and without MCI. They found decreased FC between the posterior cingulate cortex and the right temporal gyrus and increased FC between the posterior cingulate cortex and multiple brain regions in PD patients without cognitive impairment compared with healthy controls and reduced FC between the posterior cingulate cortex and several areas including bilateral prefrontal cortex, the left parietal-occipital junction, and the right temporal gyrus in PD-MCI patients compared with PD patients without cognitive impairment. The FC of the posterior cingulate cortex with other brain areas was associated with MoCA and MMSE scores in the PD patients [13]. Baggio et al. performed ICA and seed-based rs-fMRI analyses in PD patients without cognitive impairment, PD-MCI patients, and healthy controls. They found that PD-MCI patients had decreased connectivity between the dorsal attention network and the right frontoinsular regions, and this alteration was associated with attention/executive function. The DMN showed increased connectivity with medial and lateral occipito-parietal regions in PD-MCI patients, which was correlated with worse visuospatial/visuoperceptual abilities [14]. In another study by Baggio et al., graph theory-based analysis showed that PD-MCI patients had reduced long-range connections and increased local interconnectedness including higher measures of clustering, small-worldness, and modularity. The local interconnectedness was associated with visuospatial/visuoperceptual and memory functions in the PD patients [15]. Peraza et al. compared the intra- and internetwork changes in PD patients with and without MCI and found that PD-MCI patients had intranetwork impairments in the attention, executive function, and motor control networks compared with PD patients with normal cognitive function, as well as internetwork alterations in the visual perception together with the three above-mentioned networks [16]. Amboni et al. assessed the brain networks using ICA analysis in PD patients with and without MCI. Both PD groups showed impaired DMN connectivity, while the PD-MCI group showed impaired FC in the frontoparietal network. In addition, the decreased prefrontal cortex connectivity was associated with memory, visuospatial, and executive function [17]. Shin et al. compared the FC of PD-MCI patients with shorter and longer periods of motor symptoms before cognitive impairment using seed-based analyses and found that these two groups showed different characteristics of decreased FC in the DMN. Their findings implied that these two types might have different mechanisms and might help predict cognitive decline in future studies [18]. Lopes et al. investigated the brain network features of PD patients with different levels of cognitive function using the graph theory and network-based statistics. They showed that the functional organization decreased in accordance with the degree of cognitive impairment, and PD patients with cognitive impairment had reduced FC in the basal ganglia, ventral prefrontal, parietal, temporal, and occipital cortices [19]. The above studies confirmed the commonly impaired cognitive domains in PD, executive, attention, visuospatial function, and memory [7], and uncovered the impaired brain regions.

### 2.2. rs-fMRI Study on PD Patients without Cognitive Impairment

rs-fMRI is a sensitive imaging modality that can reveal dysfunction in cognition-related brain regions in PD patients with only subtle cognitive changes or even without cognitive symptoms. This property makes rs-fMRI a promising tool for the early identification of patients with a high risk for PDD. Lucas-Jimenez et al. used a seed-based FC analysis and showed reduced DMN FC in nondemented PD patients, and this FC change was correlated with lower verbal and visual memory and visual abilities in PD [20]. Manza et al. also used a seed-based approach to investigate the striatum FC in PD patients, the results showed that the stronger FC between the dorsal caudate and the rostral anterior cingulate cortex was associated with cognitive dysfunction (especially memory and visuospatial function) [21]. Muller-Oehring utilized a seed-based rs-fMRI and task fMRI and demonstrated that stronger putamen-medial parietal and pallidum-occipital FC than controls was associated with executive function and motor symptoms [22]. Tessitore et al. assessed the brain FC of cognitively unimpaired PD patients using the ICA analysis and found decreased FC within the DMN, and the FC changes correlated with memory, visuospatial, and attention/executive function [23]. Madhyastha et al. showed impaired brain network dynamic connectivity at rest in PD patients without cognitive impairment using factor analysis of overlapping sliding windows, and the factors in the dorsal attention network and frontoparietal task control network were correlated with patients' performance in attention examinations [24]. Luo et al. performed a graph theory-based analysis in drug-naive PD patients and found disrupted network organization in the PD patients at global, nodal, and connectional levels. Node centralities and connectivity strength were reduced mainly in the temporal-occipital and sensorimotor regions. Furthermore, the changed global network properties were associated with cognitive function [25]. In a group of rigidity-dominant drug-naive PD patients without cognitive impairment using a seed-based approach, Hou et al. found a decreased FC in DMN (especially the posterior DMN) and an increased FC in the anterior DMN in the PD patients, and increased FC of the anterior and ventral parts were negatively correlated with Hopkins verbal learning test-revised scores [26]. In a 3-year longitudinal study by Olde Dubbelink et al., the multivariate exploratory linear optimized decomposition into independent components analysis showed widespread reduction of FC in the PD patients compared with the controls. After 3 years, the FC in the PD patients decreased further on, and the FC changes were most prominent for posterior parts of the brain. The FC change over time was correlated with the alteration of global cognitive function, as well as perception, praxis and the spatial span subscores [27]. It is noteworthy that some patients in their study had dementia, and the cognitive performance of the PD patients was inferior to the controls at baseline [7]. In a study by Huang et al., left onset PD patients had worse performance in feedback-based associative learning than the right onset PD patients and the controls. In the left onset PD patients, the impaired cognitive function was associated with the ReHo in the right dorsal rostral putamen [28]. These studies showed that rs-fMRI was a sensitive tool detecting brain network abnormalities in PD patients without obvious cognitive impairment, and some motor symptom features implied higher risk for cognitive dysfunction in PD.

## 3. Combining rs-fMRI with Other Modalities

Until now, various methods have been applied in investigating neurological disorders, and each has its advantage and disadvantage. PET and SPECT using different radio ligands can display abnormalities of neurotransmitters in the brain and deposition of aggregated proteins such as *α*-synuclein, *β*-amyloid, and tau proteins in neurological disorders [29]. EEG has a very high temporal resolution. CSF laboratory examinations are able to detect the culprit protein and the degree of neurodegeneration [30]. Combining rs-fMRI and other modalities can deepen our understanding of the pathophysiology of cognitive dysfunction in PD. Madhyastha et al. used the “network kernel analysis” in PD patients and found widespread alterations in the correlations of network kernels in the PD patients, and the degree of network disturbance was associated with lower cerebrospinal fluid *α*-synuclein and amyloid-*β*_42_ levels. In addition, increased correlation of the insula with the DMN was associated with worse attentional function. Therefore, both *α*-synuclein and amyloid-*β*_42_ might play a role in disrupting cognitive-related brain regions [30]. Lebedev et al. combined a graph theory-based rs-fMRI analysis with ^123^I-FP-CIT SPECT imaging. In their study, executive function was associated with dorsal frontoparietal cortical processing and sensory involvement, as well as the striatal dopamine transporter binding ratios. Memory performance was correlated with prefrontolimbic processing but not associated with nigrostriatal dopaminergic function. Their study confirmed that distinct from executive dysfunction, memory deficits in PD was not induced by dopaminergic insufficiency [31]. In future studies, integration of more CSF laboratory examinations, EEG/fMRI, and PET imaging utilizing more radio ligands including for other transmitters (cholinergic, serotonergic, norepinephrinergic systems, etc.) and abnormal proteins (such as *α*-synuclein, *β*-amyloid, and tau proteins) and laboratory examinations using other body fluids can promote our recognition of the underlying mechanism of cognitive impairment in PD.

## 4. rs-fMRI as a Diagnostic Tool

So far, most of the rs-fMRI studies in PD enrolled small numbers of patients. However, we can obtain preliminary consistent conclusions on the networks disrupted in PD and their relationship with cognitive dysfunction. We still need more evidence to apply rs-fMRI as a diagnostic tool in clinical practice. Abos et al. used a support vector machine method to distinguish PD patients with and without MCI with an accuracy of 80%, and the connectivity of the selected edges was correlated with memory and executive function in the PD patients [32]. Peraza et al. compared the brain network between patients with dementia with Lewy bodies and PDD using seed-based analyses. Their results implied that there might be subtle differences in attention and motor-related networks between these two disorders [33]. Borroni et al. performed rs-fMRI in patients with PD, PDD, and DLB using ReHo. PD and PDD patients had decreased ReHo in the frontal regions, while DLB patients had lower ReHo in the posterior regions [34]. Future studies enrolling large samples would pave the road for clinical diagnostic applications in PD.

## 5. Prediction of Cognitive Impairment in PD

Disease-modifying therapies targeting *α*-synuclein, *β*-amyloid, and tau proteins are under active investigation and are hopeful to be available in clinical practice in the near future. Early identification of PD patients with a high risk for dementia is critical for potential disease-modifying therapies. As far as we know, most of the studies using rs-fMRI for cognitive function in PD are cross-sectional. Only two studies explored the progression of cognitive impairment and brain network changes in a longitudinal design. However, they only enrolled small numbers of patients and had a short time of follow-up (1 and 3 years, resp.) [21, 27]. Ongoing prospective studies such as the Parkinson's Progression Markers Initiative (PPMI) would help answer the question of PDD prediction.

## 6. Evaluating and Assisting Intervention

rs-fMRI has been applied in the assessment of the effects of levodopa and cognitive rehabilitation in PD cognition. Simioni et al. tested the effect of levodopa on brain networks and cognitive function using seed-based and ICA analyses. Levodopa increased resting state FC between caudate and right parietal cortex (within the frontoparietal attentional network), and this effect was associated with improvement in working memory performance [35]. Diez-Cirarda employed a seed-based rs-fMRI analysis in a randomized controlled trial of cognitive rehabilitation in PD and showed increased FC between the left inferior temporal lobe and the bilateral dorsolateral prefrontal cortex in the rehabilitation group than the control group. Moreover, the increased FC was correlated with executive function in the treatment group [36]. Eighteen months later, they performed a follow-up examination of 15 patients in the rehabilitation group and found preserved effect of rehabilitation on both cognitive function and brain FC even after 18 months [37]. Cerasa et al. also evaluated the effect of cognitive rehabilitation using rs-fMRI. They employed ICA and SPM and showed improved attention/executive function together with the attention and central executive neural networks [38]. There are other studies evaluating the effect of varied interventions on the resting brain networks, but the associations with cognitive function has been less investigated [39–45]. In the future, rs-fMRI can play a more important role in evaluating treatment effects as well as guiding neuromodulation therapies.

In summary, rs-fMRI might be a useful tool for the exploration of underlying mechanism of cognitive dysfunction in PD and can help diagnose cognitive dysfunction in PD and assist treatments. More longitudinal studies using rs-fMRI and the combination of rs-fMRI with other modalities are needed.
